# Whole-Lesion Histogram Analysis of the Apparent Diffusion Coefficient as a Quantitative Imaging Biomarker for Assessing the Level of Tumor-Infiltrating Lymphocytes: Value in Molecular Subtypes of Breast Cancer

**DOI:** 10.3389/fonc.2020.611571

**Published:** 2021-01-08

**Authors:** Wen-jie Tang, Zhe Jin, Yan-ling Zhang, Yun-shi Liang, Zi-xuan Cheng, Lei-xin Chen, Ying-ying Liang, Xin-hua Wei, Qing-cong Kong, Yuan Guo, Xin-qing Jiang

**Affiliations:** ^1^ Department of Radiology, Guangzhou First People’s Hospital, School of Medicine, South China University of Technology, Guangzhou, China; ^2^ Department of Ultrasound, The Third Affiliated Hospital, Sun Yat-Sen University, Guangzhou, China; ^3^ Department of Pathology, Guangzhou First People’s Hospital, School of Medicine, South China University of Technology, Guangzhou, China; ^4^ Department of Radiology, The Third Affiliated Hospital, Sun Yat-Sen University, Guangzhou, China

**Keywords:** tumor-infiltrating lymphocytes, breast cancer, apparent diffusion coefficient, magnetic resonance imaging, molecular subtypes

## Abstract

**Purpose:**

To assess whether apparent diffusion coefficient (ADC) metrics can be used to assess tumor-infiltrating lymphocyte (TIL) levels in breast cancer, particularly in the molecular subtypes of breast cancer.

**Methods:**

In total, 114 patients with breast cancer met the inclusion criteria (mean age: 52 years; range: 29–85 years) and underwent multi-parametric breast magnetic resonance imaging (MRI). The patients were imaged by diffusion-weighted (DW)-MRI (1.5 T) using a single-shot spin-echo echo-planar imaging sequence. Two readers independently drew a region of interest (ROI) on the ADC maps of the whole tumor. The mean ADC and histogram parameters (10^th^, 25^th^, 50^th^, 75^th^, and 90^th^ percentiles of ADC, skewness, entropy, and kurtosis) were used as features to analyze associations with the TIL levels in breast cancer. Additionally, the correlation between the ADC values and Ki-67 expression were analyzed. Continuous variables were compared with Student’s t-test or Mann-Whitney U test if the variables were not normally distributed. Categorical variables were compared using Pearson’s chi-square test or Fisher’s exact test. Associations between TIL levels and imaging features were evaluated by the Mann-Whitney U and Kruskal-Wallis tests.

**Results:**

A statistically significant difference existed in the 10^th^ and 25^th^ percentile ADC values between the low and high TIL groups in breast cancer (*P*=0.012 and 0.027). For the luminal subtype of breast cancer, the 10^th^ percentile ADC value was significantly lower in the low TIL group (*P*=0.041); for the non-luminal subtype of breast cancer, the kurtosis was significantly lower in the low TIL group (*P*=0.023). The Ki-67 index showed statistical significance for evaluating the TIL levels in breast cancer (*P*=0.007). Additionally, the skewness was significantly higher for samples with high Ki-67 levels in breast cancer (*P*=0.029).

**Conclusions:**

Our findings suggest that whole-lesion ADC histogram parameters can be used as surrogate biomarkers to evaluate TIL levels in molecular subtypes of breast cancer.

## Introduction

Breast cancer is the most common cancer and causes cancer-related death in women worldwide. Clinical decision-making is strictly focused on evaluating breast tumor cells and is based on assessing hormone receptors and the human epidermal growth factor receptor 2 (Her-2) status using a combination of immunohistochemical and *in situ* hybridization techniques (1). However, we are increasingly recognizing that certain cellular components in the stroma, particularly immune cells, may influence prognosis and even predict response to specific treatments. Available evidence suggests that tumor-infiltrating lymphocytes (TILs) are important and clinically meaningful, as their abundance in the intratumoral stroma strongly correlates with prognosis (2).

TILs are immune cells that have been observed in many solid tumors, including breast cancer. Some recent studies have shown that TILs represent a surrogate for a pre-existing favorable host antitumor activated T cell response (3, 4). TILs are associated with prognosis as well as response to neoadjuvant chemotherapy and immunotherapy in breast cancer (5). The International Immuno-oncology Biomarker Working Group has incorporated a standardized TIL scoring system into the guidelines (6, 7). A recent publication demonstrated the feasibility of applying a web-based TIL scoring platform to enable the use of TILs as a stratification factor in an immunotherapy clinical trial for breast cancer within a risk-management framework (8). This pilot study proposes that TIL scores can be used in the standardized workflow of future clinical trials.

The immune infiltrate and its clinical significance may differ among the molecular subtypes of breast cancer. Denkert et al. (9) showed that in luminal tumors, low TIL levels (<10%) were associated with improved overall survival (OS) and speculated that high TIL levels in ER-positive tumors might be linked to more aggressive features and/or be associated with endocrine resistance. However, in non-luminal subtypes, pre-existing immune infiltrates appear to be linked with good outcomes, where high TIL levels predict better survival and a high likelihood of achieving a pathologic complete response (pCR) (10). Therefore, it is necessary to discuss TIL levels in different molecular subtypes of breast cancer.

Despite many efforts to evaluate standardized TILs, the process remains arduous and subjective, and internal variability exists, which led to TIL assessment being limited in daily practice in many countries. Imaging-based biomarkers offer a noninvasive whole-body evaluation of tissue biomarkers, bypassing spatial heterogeneity issues, and certain sequences provide quantitative values. In addition, tumor biology is subject to change over time, and treatment may lead to changes in the tumor immune microenvironment. Therefore, imaging-based biomarkers could be very useful for noninvasive and whole-body quantification of the expression of immune-related parameters (11). They offer the advantage of serial evaluations and longitudinal measurements (before and after treatment) and enable spatial and temporal heterogeneity visualization (11). Recently, an increasing number of studies have focused on ultrasonography (US) (12), MRI (12–14), and PET (15) to assessed the correlation of imaging features and TIL levels. However, many imaging features in these studies are based on subjective judgments and lack objective quantitative indicators. It is known that the apparent diffusion coefficient (ADC) value can be evaluated objectively and quantitatively. Fogante et al. (16) reported that the ADC of samples with high TIL levels is higher than that of those with low TIL levels and speculated the ADC may play an important role in assessing TIL levels. However, the ADC measurements in the above study were performed using a manually drawn ROI from a single representative slice of the ADC map that might have a limited ability to reflect the actual whole-tumor characteristics.

In whole-lesion histogram analysis of the ADC, a volumetric ROI is positioned on the entire lesion over contiguous slices, and a histogram of ADC values reflecting the frequency of voxels is constructed, leading to better evaluation of heterogeneity. Recent studies have suggested that whole-lesion histogram analysis of the ADC might have additional value when assessing the heterogeneity and aggressiveness of breast cancer (17, 18). A growing number of studies have used ADC histogram parameters as a potential imaging biomarkers for predicting histopathological features in different tumors, such as Ki-67 (19, 20), EGFR (21, 22), the hormone receptor status (23, 24), and some significant results have been obtained. Likewise, if it is possible to assess the TIL levels from ADC histogram parameters, it may help predict the prognosis and an effective treatment strategy.

Therefore, this study aimed to investigate possible associations between quantitative ADC metrics derived from whole-lesion histogram analysis and the TIL levels, specifically in the different molecular subtypes of breast cancer.

## Materials and Methods

### Patients

**Figure 1:**
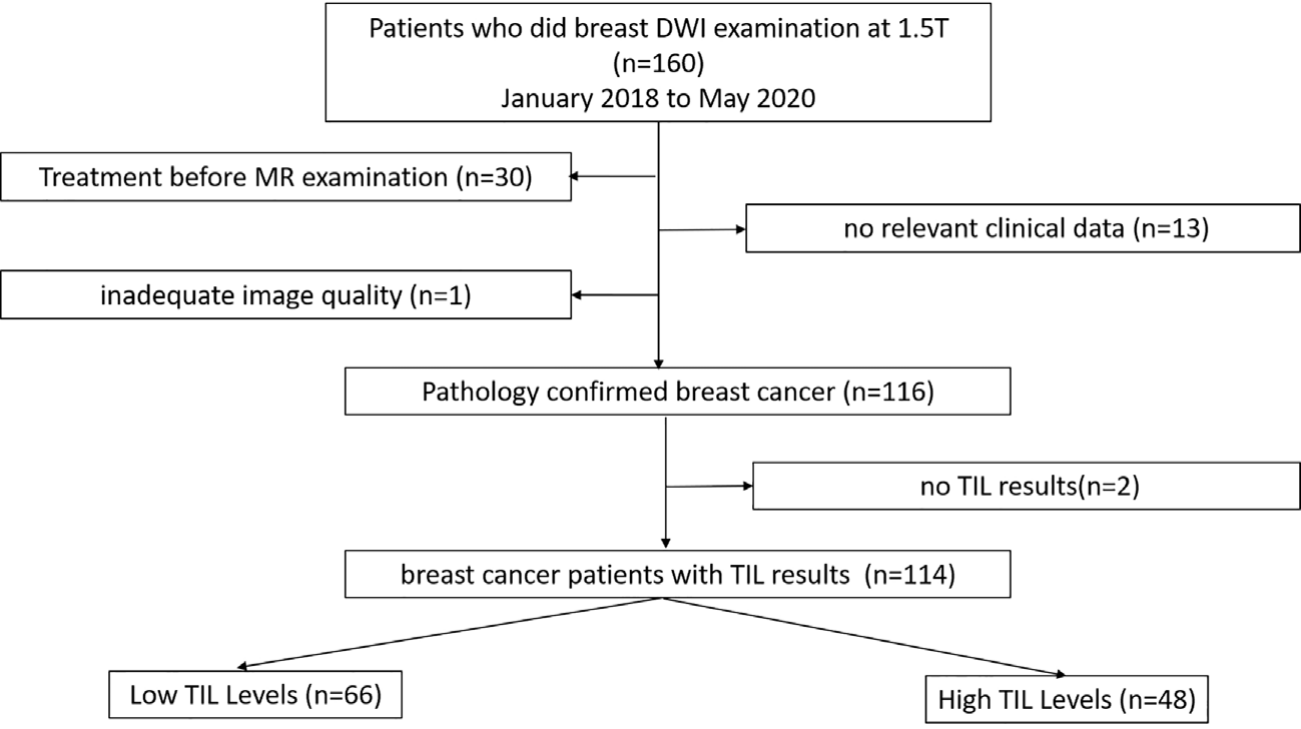
Flow chart of the patient selection towards study cohort.

This retrospective study was conducted under the approval of the Ethics Committee of the Second Affiliated Hospital of South China University of Technology. Between January 2018 and May 2020, 160 patients with suspicious findings on mammography or ultrasound underwent breast MRI at our institution. One hundred fourteen patients who fulfilled the following inclusion and exclusion criteria were evaluated. The inclusion criteria were as follows: (1) patients with pathologically diagnosed breast cancer after surgery; (2) patients who underwent standard breast magnetic resonance imaging, whose results included axial T1-weighted images, fat-suppressed T2-weighted images, and axial fat-saturated T1-weighted images pre- and post-enhancement and diffusion-weighted imaging (DWI) sequences; and (3) patients who had complete relevant clinical data. The exclusion criteria were as follows: (1) breast-related treatment before MRI; (2) no relative clinical information; and (3) inadequate image quality. The patient selection process is demonstrated in **Figure 1**.

### MR Examination Protocol

One hundred fourteen patients underwent breast MR imaging examinations using a 1.5-T system [uMR 560 1.5 T scanner (United Imaging, Shanghai, China)] and a dedicated four-channel SENSE breast coil. The patients were placed in the prone position with the breasts immobilized. The MRI acquisition protocols were standardized as follows. First, transverse T1-weighted and fat-suppressed T2-weighted images were obtained. Second, transverse DWI was performed using a single-shot spin-echo echo-planar imaging sequence with the following parameters: repetition time/echo time (TR/TE), 3800/78 ms; matrix, 156×156; slice thickness, 4 mm; slice number, 27; voxel size: 2.0×2.0×4.0 mm^3^; b value: 50 and 800 s/mm^2^; number of averages, 1; acquisition time, 103 s. Third, the gadolinium-based agent gadopentetate dimeglumine (Gd-DTPA, Magnevist; Bayer Healthcare, Berlin, Germany) was intravenously injected at a dose of 0.2 ml/kg of body weight at a rate of 1.5 ml/s, followed by a 20-ml saline flush performed with a high-pressure injector. Axial 3D fat-saturated T1-weighted images were obtained immediately before contrast administration and at six consecutive time points following the administration of the Gd-DTPA contrast agent, with the following parameters: TR/TE, 5.1/2.1 ms; flip angle, 10; matrix, 400×70. ADC maps were generated with a monoexponential fit for the diffusion data with b values of 50 and 800 s/mm^2^ using the following formula: ADC=[lnS0−lnS(b)]/b [where S0 and S(b) represent the DWI signal intensity at b=50 and 800 s/mm^2^, respectively].

### Imaging Analysis

**Figure 2:**
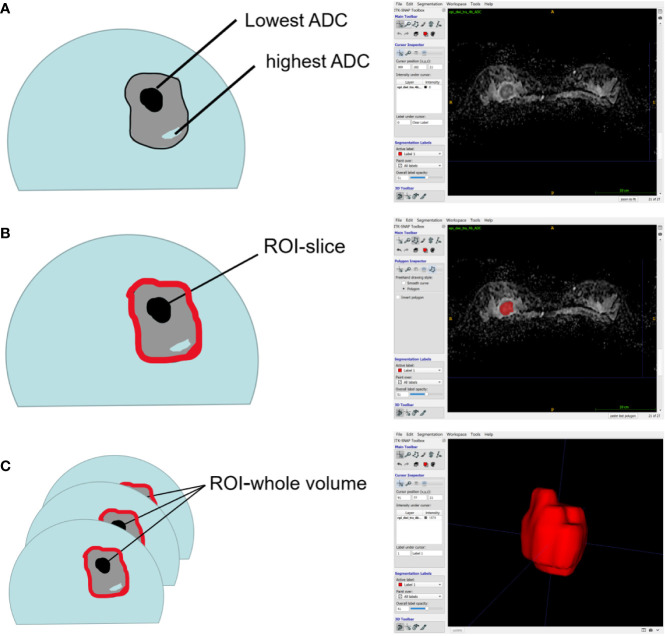
Schematic representation of the ROI-placement approaches. Image **(A)** shows a breast lesion with heterogeneous ADC-values. The ROI (red lines) placement approaches were as follows: a 2D ROI covering the whole lesion on one slice **(B)**; and a 3D- ROI covering the whole lesion on all slices **(C)**.

All DWI scans were retrospectively reviewed by two radiologists (Reader 1, with 12 years of experience in breast MRI; and Reader 2, with 6 years of experience in breast MRI). The radiologists were blinded to the histopathological results. The references for tumor detection were the dynamic contrast-enhanced images and axial T2-weighted images. Whole volume ROI placement approaches were applied as multiple large 2D ROIs were manually drawn on each slice containing the whole lesion of interest and were then combined to create a 3D ROI using ITK-SNAP (3.8.0) (**Figure 2**). The whole volume ADC histogram, including any cystic or necrotic portions and hemorrhagic components, was evaluated to assess the heterogeneity of the tumor. The analysis was performed using Python (3.8.6). An ROI containing the whole tumor generated an entire tumor volume reconstruction, and the calculated results were displayed in the form of a histogram with the matplotlib package in Python. Various ADC histogram parameters were calculated: 10^th^ percentile, mean, 50^th^ percentile (median), 90^th^ percentile, skewness (a measure of the asymmetry of the histogram about its mean), kurtosis (a measure of the peakedness of the histogram) and entropy (measure of the variation in the histogram distribution).

### Pathology

Pathological data, including tumor size, grade, and immunohistochemical (IHC) marker status, were extracted from pathology reports. The evaluated pathological data included ER, PR, and Her-2 expression and the Ki-67 index. All cases were divided into luminal (luminal A and luminal B) and non-luminal subtypes (Her-2-overexpressing and triple-negative breast cancer). The Ki-67 index was determined and used to classify patients into a low-Ki-67 level (Ki-67 < 14%) and a high Ki-67-level group (Ki-67≥14%).

**Figure 3:**
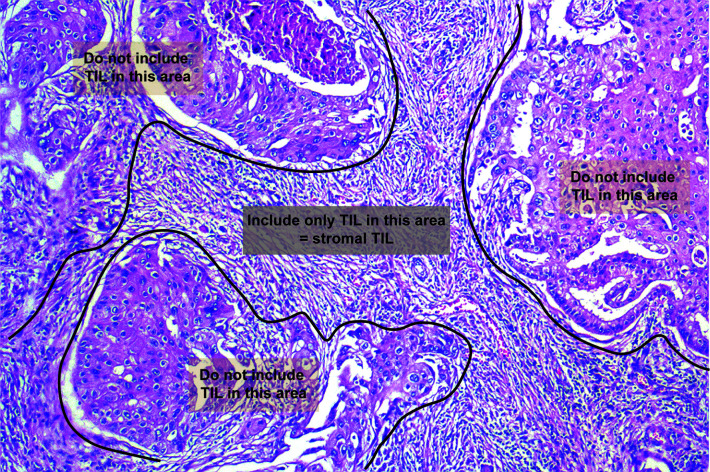
Schematic representation of TILs assessment in the stromal compartment.

Histologically, TILs in the stromal compartment were assessed and analyzed according to the International TIL Working Group (6) (**Figure 3**). TIL evaluation was blinded to the imaging results. For evaluating TILs, the boundaries of the tumor should be identified with only TILs inside them evaluated. TILs in areas with crush artefacts, necrosis, and inflammation around biopsy sites or extensive central regressive hyalinization should not be scored. A necrotic biopsy is considered unscorable. The ratio of the lymphoid cells to stroma within the tumor was recorded as a percentage, and were classified into three categories: <10, 10–50, and >50%. To facilitate statistical analysis, we defined samples with less than 10% TILs as low TIL levels and samples with 10% or more as high TIL levels (9).

### Statistical Analysis

Statistical analysis was performed using SPSS 21.0 (IBM Corp., Armonk, NY, USA) and MedCalc 8 (MedCalc Software, Ostend, Belgium). Interobserver agreement was evaluated between the two observers. The interobserver agreement of the analysis between the two radiologists was evaluated by calculating the ICC. The ICCs were interpreted according to the criteria of Landis and Koch (25): 0.00 to 0.20, poor agreement; 0.21 to 0.40, fair agreement; 0.41 to 0.60, moderate agreement; 0.61 to 0.80, good agreement; and 0.81 to 1.00, excellent agreement. Averaged histogram results of the two radiologists were used for further analysis. Continuous variables were compared with Student’s t-test or Mann-Whitney U test if the variables were not normally distributed. Categorical variables were compared using Pearson’s chi-square test or Fisher’s exact test. Associations between TIL levels as well as Ki-67 levels and imaging features were evaluated by the Mann-Whitney U and Kruskal-Wallis tests. Based on the significant variables acquired from the univariate analysis, multivariate logistic regression analysis was performed.

## Results

### Patient Characteristics

Sixty-six (57.89%) of 114 lesions had low TIL levels, and 48 (42.11%) had high TIL levels. Of these 114 tumors, 85 (74.5%) were invasive ductal carcinomas, 14 (12.3%) were invasive lobular carcinomas, 9 (7.9%) were mixed ductal and lobular carcinomas, and 6 (5.3%) were micropapillary carcinomas. Tumors with high TIL levels had significantly higher Ki-67 expression than those with low TIL levels (*P* = 0.007). There was a statistically significant difference between molecular subtypes and TIL levels (*P* = 0.001), as shown in **Table 1**.

**Table 1:** The histopathological results of the patient cohort.

Variable	Low TIL levels (n=66)	High TIL levels (n=48)	Statistical value	*P*-value
**Mean tumor size (mm^a^)**	29.5 ± 17.4	30.3 ± 18.0	−0.220	0.827
**Mean age (years^a^)**	52.8 ± 11.6	51.2 ± 9.3	0.777	0.439
**Tumor size**				
**≤2cm**	26	13	−1.362	0.173
**>2cm**	40	35		
**Age at diagnosis**			−0.100	0.921
**≤50**	31	23		
**>50**	35	25		
**Menopausal status**			−1.670	0.095
** premenopausal**	28	28		
** postmenopausal**	38	20		
**Histology**			−0.831	0.406
**Invasive ductal carcinoma**	55	37		
** others**	11	11		
**Axillary Lymph node**			−0.221	0.825
**negative**	48	34		
**positive**	18	14		
**Ki-67 status**			−2.677	0.007
**<14%**	21	5		
**≥14%**	45	43		
**Molecular subtypes**			−3.256	0.001
** Luminal**	50	22		
** Non-Lumnial**	16	26		

^a^Data are mean values ± standard deviations.

### TIL Level Discrimination Using Whole-Volume ADC Histogram Analysis

For the mean ADC and ADC histogram parameters, the ICC analysis showed a good agreement among the two readers with ICC values ranging from 0.801–0.835. The results demonstrated significant differences in the 10^th^ and 25^th^ ADC histogram parameters between the low and high TIL levels of breast cancers (*P*=0.012 and *P*=0.027; **Table 2**).

**Table 2:** Comparison of different parameters of the whole-volume ADC histogram in the breast cancers with low and high TIL levels.

Variable	Low TIL levels (n=66)	High TIL levels (n=48)	*t* value	*P*-value
**Mean ADC** **(×10^−6^ mm^2^/s)**	1,112.06 ± 191.90	1,190.90 ± 235.35	−1.968	0.052
**10^th^ percentile ADC (×10^−6^ mm^2^/s)**	784.38 ± 190.18	870.42 ± 161.03	−2.541	0.012
**25^th^ percentile ADC (×10^−6^ mm^2^/s)**	916.68 ± 181.05	994.79 ± 184.92	−2.254	0.027
**50^th^ percentile ADC (×10^−6^ mm^2^/s)**	1,081.42 ± 192.56	1,156.60 ± 241.09	−1.850	0.078
**75^th^ percentile ADC (×10^−6^ mm^2^/s)**	1,283.09 ± 225.82	1,356.63 ± 316.97	−1.374	0.173
**90^th^ percentile ADC (×10^−6^ mm^2^/s)**	1,494.08 ± 284.80	1,571.52 ± 340.96	−1.319	0.190
**skewness**	0.45 ± 0.60	0.61 ± 0.64	−1.357	0.178
**entropy**	6.73 ± 1.42	6.84 ± 1.38	−0.414	0.679
**kurtosis**	1.22 ± 1.53	1.81 ± 1.89	−1.848	0.067

The 10^th^, 25^th^, and 50^th^ percentile ADC values, kurtosis and mean ADC were significantly higher in the luminal subtype than in the non-luminal subtype of breast cancer (P<0.001, P=0.002, P=0.025, P=0.007 and P=0.013). Significant differences were found in the above ADC histogram parameters among different molecular subtypes of breast cancer (**Table 3**).

**Figure 4:**
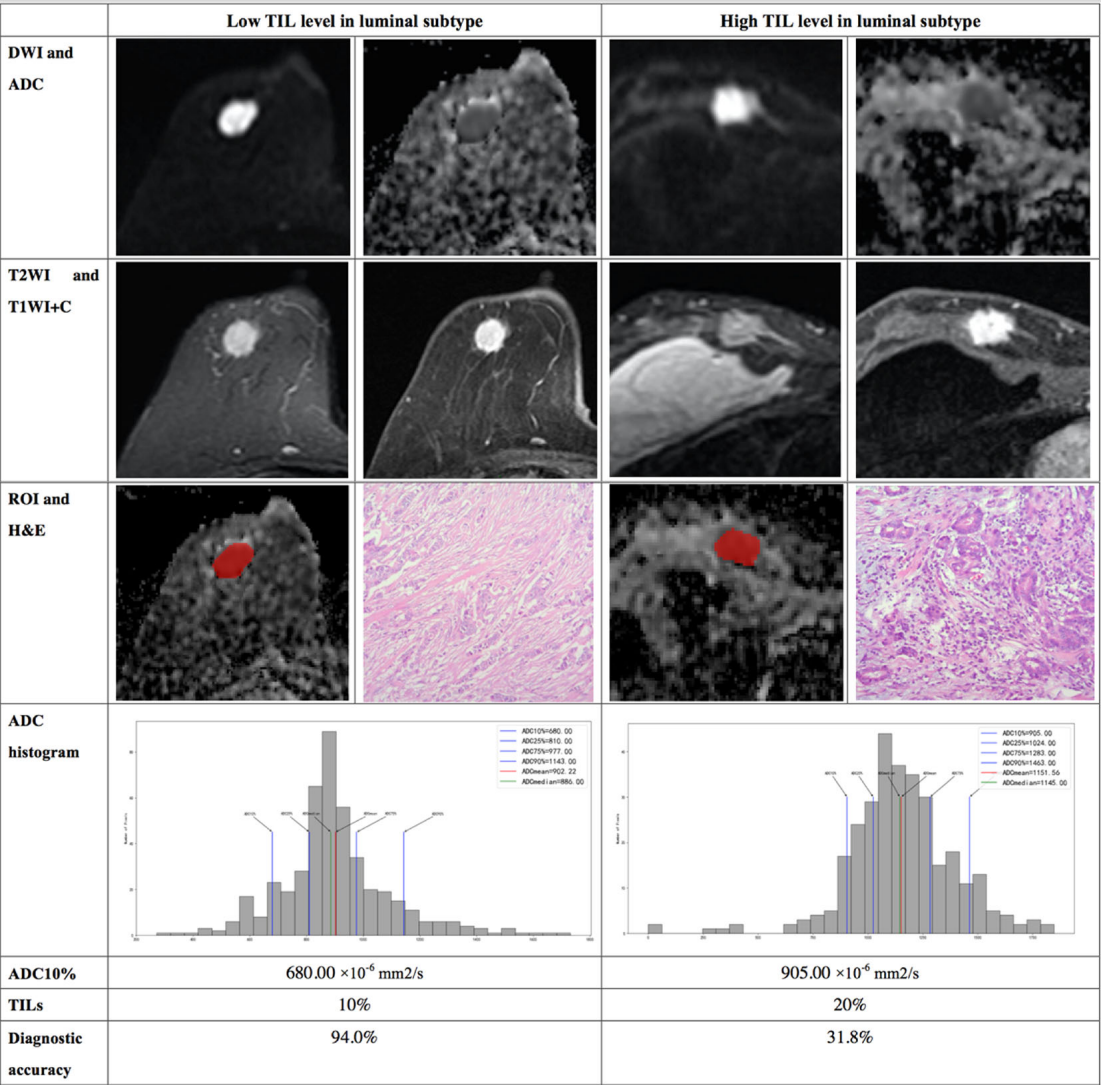
Illustration of two patients with the luminal subtype of breast cancer, who underwent magnetic resonance (MR) imaging and were assessed for tumor-infiltrating lymphocytes (TILs) from the pathologists’ reading. The DWI, ADC, T2WI, T1WI+C, ROI, H&E, and ADC histogram data were collected from the patients in each group. Additionally, the value of the 10^th^ percentile ADC and TIL level of each patient, as well as the diagnostic accuracy for the low and high levels of TIL in the luminal subtype, were introduced.

**Figure 5:**
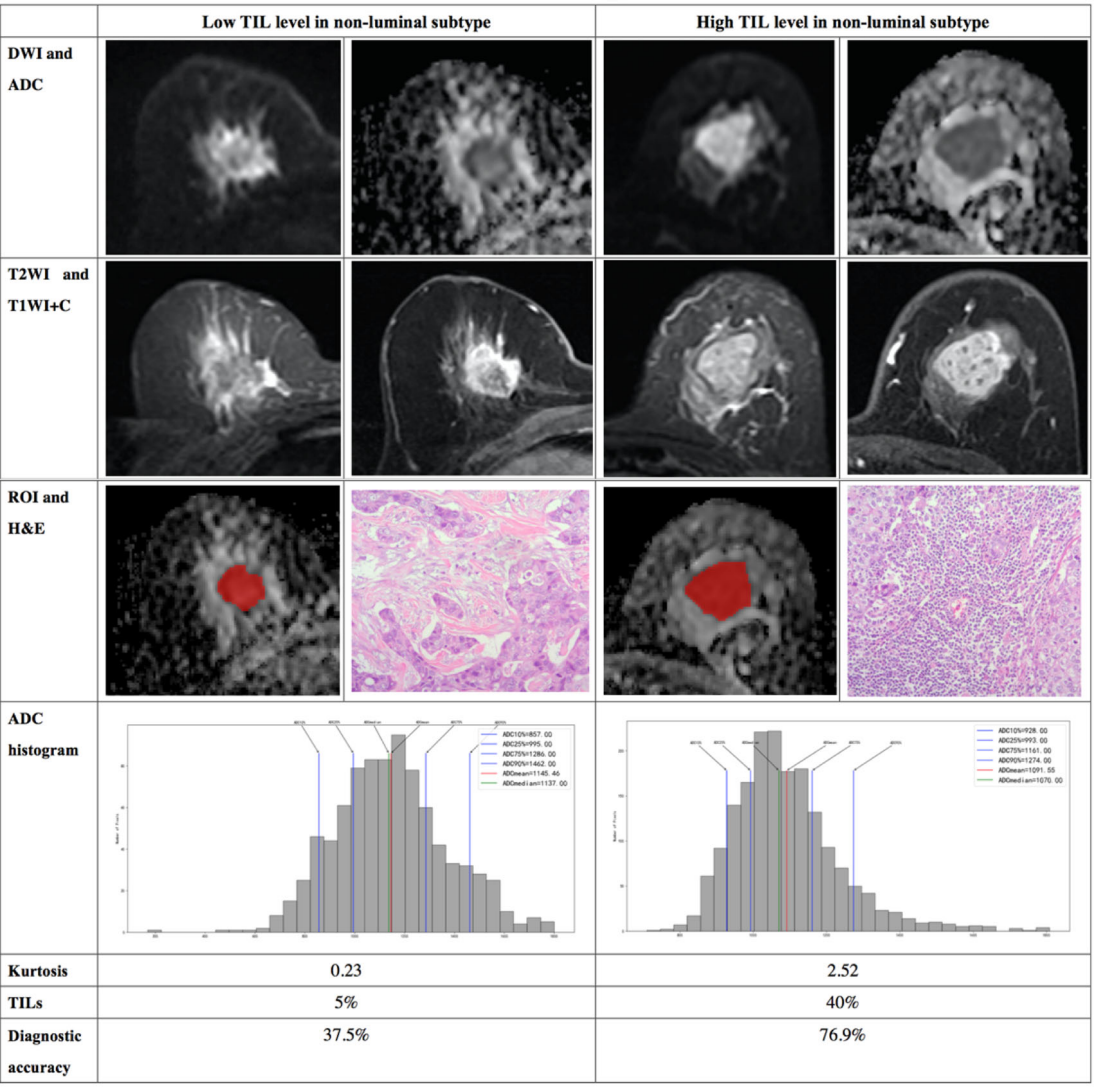
Illustration of two patients with the non-luminal subtype of breast cancer, who underwent magnetic resonance (MR) imaging and were assessed for tumor-infiltrating lymphocytes (TILs) from the pathologists’ reading. DWI, ADC, T2WI, T1WI+C, ROI, H&E, and ADC histogram data were collected from the patients in each group. Additionally, the kurtosis and TIL level of each patient, as well as the diagnostic accuracy for the low and high levels of TIL in the non-luminal subtype, were introduced.

**Figure 6:**
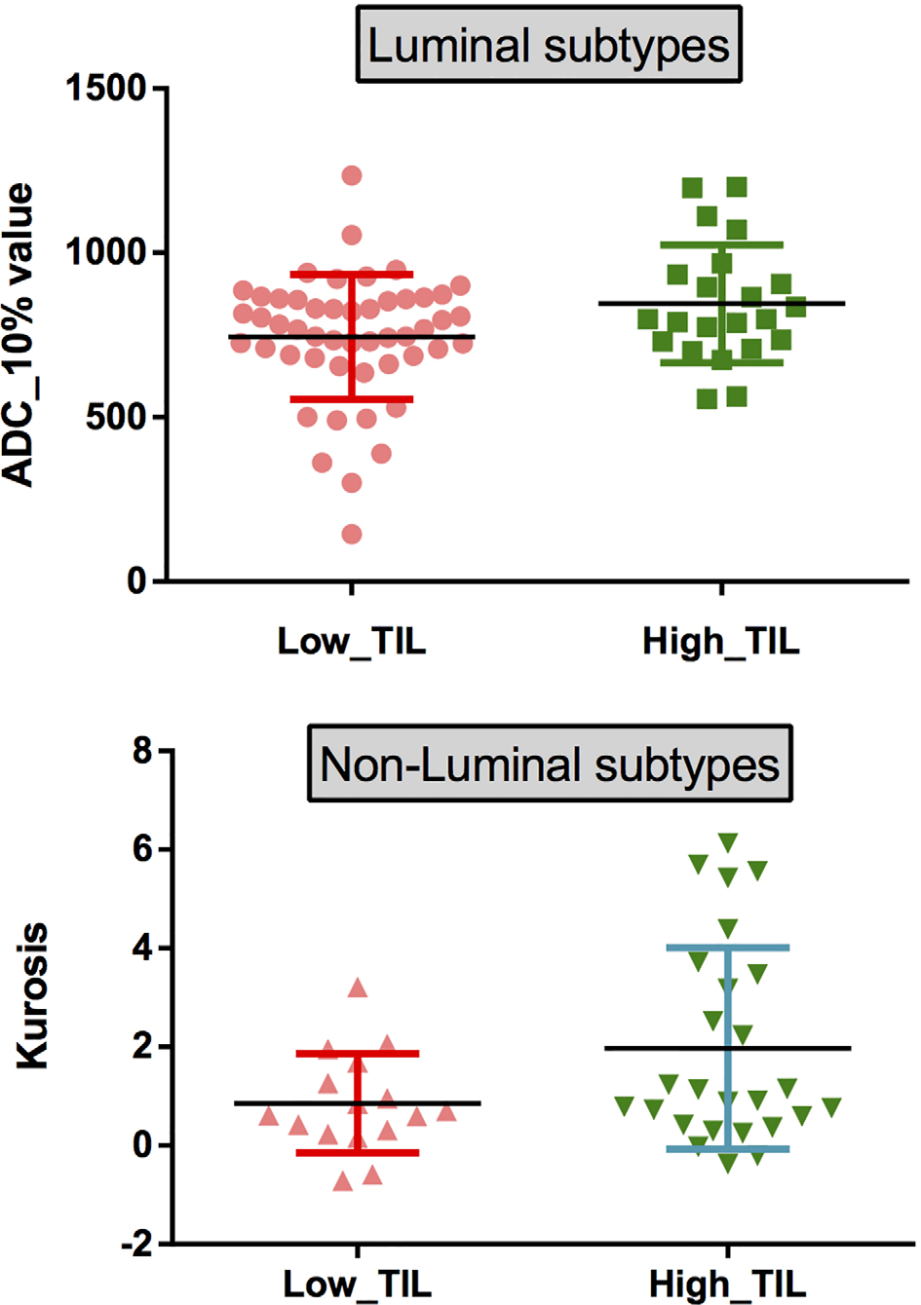
(top) Scatterplots of the 10^th^ percentile ADC values of lesions with low and high levels of TILs in patients with the luminal subtypes. (bottom) Scatterplots of the kurtosis of lesions with low and high levels of TILs in patients with the non-luminal subtypes.

The results demonstrated that the 10^th^ percentile ADC value was significantly higher for samples with high TIL levels than for those with low TIL levels in the luminal subtype of breast cancer (*P*=0.041) (**Figure 4**). The kurtosis was significantly higher for samples with high TIL levels in the non-luminal subtype of breast cancer (*P*=0.023) (**Figure 5**). However, other ADC histogram parameters did not show a significant difference (*P >*0.05) (**Table 3**). Scatterplots of the 10^th^ percentile ADC value and kurtosis of lesions with low and high TIL levels are shown in **Figure 6**.

**Table 3:** Comparisons of ADC histogram parameters according to TILs and the ER/PR/HER2 status.

	Luminal (n=72)	Non-luminal (n=42)		Luminal (n=72)			Non-luminal (n=42)		
**TIL levels**			P-value	Low level (n=50)	High level (n=22)	P-value	Low level (n=16)	High level (n=26)	P-value
**10^th^ percentile ADC (×10^−6^ mm^2^/s)**	775.07 ± 191.47	898.67 ± 136.79	<0.001	744.12 ± 188.17	845.41 ± 175.22	0.041	910.19 ± 128.14	891.58 ± 143.87	0.674
**25^th^ percentile ADC (×10^−6^ mm^2^/s)**	908.89 ± 189.57	1,019.31 ± 158.68	0.002	880.30 ± 176.09	973.86 ± 198.58	0.118	1,030.38 ± 143.13	1,012.50 ± 169.93	0.728
**50^th^ percentile ADC (×10^−6^ mm^2^/s)**	1,078.58 ± 222.23	1,172.21 ± 195.06	0.025	1,048.08 ± 184.25	1,147.91 ± 274.43	0.196	1,185.63 ± 179.54	1,163.96 ± 207.06	0.731
**75^th^ percentile ADC (×10^−6^ mm^2^/s)**	1,281.60 ± 281.84	1,369.62 ± 269.74	0.092	1,249.76 ± 221.87	1,353.96 ± 370.06	0.326	1,387.25 ± 204.00	1,358.89 ± 261.45	0.714
**90^th^ percentile ADC (×10^−6^ mm^2^/s)**	1,489.97 ± 328.38	1,589.62 ± 269.74	0.099	1,460.20 ± 295.27	1,557.64 ± 378.51	0.361	1,599.94 ± 212.35	1,583.27 ± 303.58	0.849
**skewness**	0.46 ± 0.62	0.63 ± 0.62	0.152	0.41 ± 0.0.64	0.56 ± 0.54	0.197	0.58 ± 0.41	0.66 ± 0.72	0.671
**entropy**	1.42 ± 1.67	1.55 ± 1.79	0.705	6.49 ± 1.33	6.54 ± 1.34	0.120	7.47 ± 1.46	7.09 ± 1.35	0.396
**kurtosis**	6.51 ± 1.34	7.23 ± 1.39	0.007	1.33 ± 1.64	1.62 ± 1.69	0.903	0.86 ± 1.00	1.97 ± 2.04	0.023

### Ki-67 Levels Analysis Based on Whole-Volume ADC Histogram

The results demonstrated significant differences in the skewness between the low and high Ki-67 levels of breast cancers (*P*=0.029), as shown in **Table 4**. The skewness was significantly higher for samples with high Ki-67 levels in breast cancer. While, other ADC histogram parameters showed no significant difference (*P >*0.05). Additionally, Spearman’s correlation analysis between Ki-67 expression and skewness showed a weak positive correlation (*ρ*=0.205) (**Supplement Table 1**).

**Table 4:** Comparison of different parameters of the whole-volume ADC histogram in the breast cancers with low and high Ki-67 levels.

Variable	Low Ki-67 levels (n=26)	High Ki-67 levels (n=88)	*t* value	***P*-value**
**Mean ADC** **(×10^−6^ mm^2^/s)**	1,176.95 ± 205.95	1,135.89 ± 216.44	0.86	0.392
**10^th^ percentile ADC (×10^−6^ mm^2^/s)**	814.12 ± 160.15	822.52 ± 189.76	−0.21	0.838
**25^th^ percentile ADC (×10^−6^ mm^2^/s)**	965.08 ± 147.09	944.99 ± 196.47	0.48	0.631
**50^th^ percentile ADC (×10^−6^ mm^2^/s)**	1,153.65 ± 214.27	1,101.09 ± 216.97	1.09	0.279
**75^th^ percentile ADC (×10^−6^ mm^2^/s)**	1,371.69 ± 321.67	1,297.02 ± 251.16	1.25	0.215
**90^th^ percentile ADC (×10^−6^ mm^2^/s)**	1,580.19 ± 395.55	1,510.87 ± 281.51	1.00	0.320
**skewness**	0.29 ± 0.78	0.59 ± 0.55	−2.21	0.029
**entropy**	6.41 ± 1.41	6.88 ± 1.38	−1.51	0.134
**kurtosis**	1.52 ± 1.95	1.45 ± 1.64	0.183	0855

### Multivariate Analysis

In multivariate regression analysis using the 10^th^ percentile ADC value, kurtosis, tumor size, age and Ki-67 status, we found that the 10^th^ percentile ADC value, kurtosis and Ki-67 were significant independent variables associated with TIL levels (*P* = 0.012, *P* = 0.046 and *P* = 0.007, respectively). Multivariate analysis showed that the 10th percentile ADC values, kurtosis, Ki-67, age and tumor size assessed TIL levels, and the diagnostic accuracy in the luminal subtype was up to 75% and that in the non-luminal subtype was up to 61.9%. In addition, the diagnostic accuracy for low TIL levels in the luminal subtype was up to 94%, and that for high TIL levels in the non-luminal subtype was up to 76.9% (**Table 5**).

**Table 5:** Multivariate logistic regression analysis for assessing TIL levels.

	Breast cancer			Luminal subtypes			Non-luminal subtypes		
**variables**	Adjusted OR	95% CI	*P*-value	Adjusted OR	95% CI	*P*-value	Adjusted OR	95% CI	*P*-value
**10^th^ percentile ADC**	0.003	1.000–1.006	0.012	0.003	1.000–1.007	0.049	−0.001	0.994–1.005	0.839
**Tumor size**	−0.006	0.971–1.018	0.628	−0.014	0.947–1.026	0.478	−0.006	0.960–1.028	0.717
**Age**	−0.006	0.955–1.034	0.755	−0.030	0.917–1.027	0.305	0.043	0.971–1.124	0.243
**Ki-67**	1.570	1.529–15.105	0.007	1.228	0.917–12.721	0.067	0.185	0.051–28.463	0.909
**Kurtosis**	0.260	1.005–1.673	0.046	0.130	0.814–1.591	0.448	0.485	0.957–2.760	0.073

CI, confidence interval; OR, odds ratio.

## Discussion

Our study demonstrated that, to some extent, whole-lesion histogram analysis of the ADC could be used as a quantitative imaging biomarker for assessing the TIL levels in breast cancer. The 10^th^ percentile and 25^th^ percentile ADC values were significantly different among breast cancer samples stratified by TIL levels. The 10^th^ percentile ADC values and kurtosis can be used to evaluate the TIL levels in luminal and non-luminal subtypes, respectively. The combination forecasting model including 10^th^ percentile ADC values, kurtosis, Ki-67, age and tumor size has an important value for evaluating the TIL levels. Additionally, the ADC histogram parameters also played a role in assessing the Ki-67 levels.

In this study, we found a significant difference in the 10^th^ and 25^th^ percentile ADC values between the low and high TIL levels. In the reports of Celebi et al. (12) and Fogante et al. (16), ROIs were drawn in the solid component of the tumor avoiding necrotic, cystic or hemorrhagic areas, that is, the region of the minimum ADC. They reported that the low TIL group showed significantly lower ADC values than the high TIL group. These results are concordant with our study; our study showed that the 10^th^ and 25^th^ percentile ADC values tended to be lower in samples with low TIL levels than in samples with high TIL levels.

We also found that high TIL levels were significantly more common in the non-luminal subtype than in the luminal subtype of breast cancers, and this result was consistent with previous study findings (26, 27). This result also provided strong evidence that different immunobiological infiltrates exist in different molecular subtypes and that the non-luminal subtypes of breast cancer have greater immunogenicity.

Furthermore, we found that several parameters, including the 10^th^, 25^th^, and 50^th^ percentile ADC values and kurtosis, were higher than those of the luminal subtype. Choi et al. (28) reported significant differences in the mode, 25^th^ and 50^th^ percentiles, and kurtosis between triple-negative subtypes breast cancer and the EP-positive subtype, a finding that was consistent with ours. Previous studies also demonstrated that ADC measurements derived from the entire tumor were related to the ER, PR, and HER2 status (23, 29). The result further proved that different molecular types of breast cancers might need to be discussed hierarchically to avoid the interference of heterogeneity caused by the molecular subtypes when assessing the TIL levels.

Therefore, we further conducted hierarchical research on the relationship between the ADC histograms and TIL levels in the molecular subtypes of breast cancer. In the luminal subtype, there was a statistically significant difference in the 10^th^ percentile ADC values between the low and high TL levels. Shin et al. (20) showed no significant association between ADC values and different TIL levels in patients with ER-positive breast cancer. We suspect this discrepancy is related to the sample choice. The luminal subtypes of breast cancer have relatively homogeneous histologic components, with little or no necrotic or cystic components. The region showing minimum ADC may reflect the highest cellular area within the tumor and is more representative of tumor grade or aggressiveness (30). Multivariate analysis showed that the diagnostic accuracy of the 10^th^ percentile ADC value, kurtosis, Ki-67, age and tumor size for the TIL levels of luminal subtype was up to 75.0%, with a high diagnostic accuracy. More importantly, the diagnostic accuracy for low TIL levels was up to 94%. We assume that low-percentile ADC value based on whole-lesion histogram analysis may facilitate the accurate assessment of the TIL levels in luminal subtypes of breast cancer.

Interestingly, we found that, in the non-luminal subtype, kurtosis, but not the 10^th^ percentile ADC value, is a statistically significant assessment tool of the TIL levels. Kurtosis reflects the peakedness of the histogram distribution and measures the shape of the probability distribution (31). We hypothesize that non-luminal subtype lesions with higher TIL levels may have more complex pathological heterogeneity due to cancer nests, necrosis and intraductal components, among other manifestations. This finding suggests that it may be necessary to make a differentiated assessment for patients with different molecular subtypes of breast cancer when evaluating the TIL levels using MR images. Multivariate analysis of the 10^th^ percentile ADC value, kurtosis, Ki-67, age and tumor size to assess the TIL levels showed that the high TIL levels had high diagnostic accuracy.

Additionally, the Ki-67 index showed a significant difference in breast cancer samples with different TIL levels. Ki-67 is an important factor in the synthesis of ribosomes in dividing cells (32), and one of the most reliable indicators for evaluating the degree of proliferation of malignant breast cancer cells (33, 34). Lesions with high TIL levels tend to have a significantly higher Ki-67 index than those with low levels of TILs. Therefore, we speculated that breast cancer with high TIL levels has higher degree of tumor cell proliferation.

We also evaluated the correlation between the ADC parameters and the Ki-67 level in breast cancer. Our results indicated higher skewness in lesions with a high Ki-67 level than those with a low Ki-67 level. Skewness, a measure of asymmetry of the probability distribution of a histogram pattern, has been discussed regarding its value in evaluating the prognosis and efficacy of malignant tumors (35, 36). Previous studies (37–39) have reported that the mean ADC value is not or weakly correlated with Ki-67 expression, which cannot be used as a surrogate marker for proliferation activity in breast cancer. These results were consistent with our study findings. Our analysis may be related to the limited information obtained from the conventional methods of using minimum or mean ADCs in the above studies. In addition, significant differences were found in the ADC parameters using whole-lesion histogram analysis, further demonstrating that this method provided additional information (19). However, our results revealed a weak correlation between Ki-67 expression and ADC histogram parameter. Further studies with a larger sample size and multiple centers are needed to obtain definitive results.

This study has several limitations. First, this was a single-center retrospective study, which may have limited the universality of the findings. Therefore, our results need to be validated by independent, ideally prospective, studies. Additionally, in our study, the number of patients included was limited, particularly for the non-luminal subtype. Second, we only focused on the whole-lesion ADC histogram to discriminate the TIL levels. Combined with other imaging modalities such as T2-weighted and dynamic contrast-enhanced (DCE)-MRI, may be incorporated in future studies. Third, spatial incongruencies may exist between radiology and histology because the ADC maps were performed as a whole-lesion measurement and the TIL assessment only focused on a part of the tumor. Finally, because of the short follow-up time, this study lacked prognostic information, and longitudinal follow-ups are warranted in a future study.

In conclusion, whole-lesion ADC histograms can be a quantitative imaging tool for discriminating different TIL levels. Assessment using whole-lesion histogram analysis of the ADC could play a role in evaluating the TIL levels in molecular subtypes, allowing therapies to be tailored and adjusted for patients with different molecular subtypes of breast cancer.

## Data Availability Statement

The raw data supporting the conclusions of this article will be made available by the authors, without undue reservation.

## Ethics Statement

This retrospective study was conducted under the approval of the Ethics Committee of the Second Affiliated Hospital of South China University of Technology.

## Author Contributions

WT and YG designed the study and writted original draft. ZJ and YZ collected and analyzed data. YL analyzed and explained the pathology. ZC and LC did statistical analysis. YL and XW did formal analysis. QK and XJ made multiple revisions to the manuscript. All authors contributed to the article and approved the submitted version.

## Funding

This study was supported by National Natural Science Foundation of China (No. 81901711).

## Conflict of Interest

The authors declare that the research was conducted in the absence of any commercial or financial relationships that could be construed as a potential conflict of interest.
