# Supplementary material for: Whole-Lesion Histogram Analysis of the Apparent Diffusion Coefficient as a Quantitative Imaging Biomarker for Assessing the Level of Tumor-Infiltrating Lymphocytes: Value in Molecular Subtypes of Breast Cancer
Source: Front Oncol. 2021 Jan 8;10:611571. doi: 10.3389/fonc.2020.611571 (PMC7820903; doi:10.3389/fonc.2020.611571)
Supplement: Supplementary file 1 [file Table_1.docx]

Supplement Table 1: Spearman Correlations between the analyzed ADC histogram parameter and Ki-67 level

| Variable | Ki-67 | |
| --- | --- | --- |
|  | *ρ* | *P-*value |
| Mean ADC  (×10^-6^ mm^2^/s) | -0.075 | 0.428 |
| 10^th^ percentile ADC (×10^-6^ mm^2^/s) | -0.026 | 0.783 |
| 25^th^ percentile ADC (×10^-6^ mm^2^/s) | -0.067 | 0.481 |
| 50^th^ percentile ADC (×10^-6^ mm^2^/s) | -0.095 | 0.316 |
| 75^th^ percentile ADC (×10^-6^ mm^2^/s) | -0.093 | 0.323 |
| 90^th^ percentile ADC (×10^-6^ mm^2^/s) | -0.058 | 0.539 |
| skewness | 0.205 | 0.029 |
| entropy | 0.120 | 0.204 |
| kurtosis | 0.025 | 0.791 |
